# *Lactobacillus plantarum* LP45 inhibits the RANKL/OPG signaling pathway and prevents glucocorticoid-induced osteoporosis

**DOI:** 10.29219/fnr.v67.9064

**Published:** 2023-03-24

**Authors:** Xiaofeng Jiang, Xiaojun Qi, Chao Xie

**Affiliations:** 1 Department of Joint Surgery, The Affiliated Yantai Yuhuangding Hospital of Qingdao University, Yantai, China; 2 Department of Spine Surgery, The Affiliated Yantai Yuhuangding Hospital of Qingdao University, Yantai, China

**Keywords:** osteoporosis, probiotics, inflammation, gut microbiota, bone

## Abstract

**Objective:**

To examine the potential effect of the probiotic strain *Lactobacillus plantarum* LP45 on osteoporosis and to explore the involved molecular mechanisms.

**Methods:**

A rat model of glucocorticoid-induced osteoporosis (GIO) was established, which was also orally administered with increasing doses of LP45 for 8 weeks. After the termination of the 8-week treatment, the tibia and femur bones of rats were analyzed for bone histomorphometry, bone mineral content (BMC), and bone mineral density (BMD). Femoral biomechanics were assessed. In addition, levels of osteocalcin, tartrate-resistant acid phosphatase 5 (TRAP5), osteoprotegerin (OPG), and receptor activator of nuclear factor kappa-B ligand (RANKL) in the serum and bone marrow were also measured using ELISA, Western blot, and real time-polymerase chain reaction.

**Results:**

GIO caused obvious defects in tibia and femur bone structures, in terms of tissue/bone volume, trabecular separation, trabecular thickness, and trabecular number, which could be rescued by LP45 dose dependently. The GIO-induced reductions in BMC, BMD, osteoblast surfaces per bone surface (BS), as well as elevated osteoclast surface per BS were largely restored by LP45 administration dose-dependently. LP45 also increased femoral biomechanics of GIO rats. Importantly, LP45 dose-dependently restored the changes of osteocalcin, TRAP5, OPG, and RANKL in the serum as well as bone marrow of GIO rats.

**Conclusion:**

Oral LP45 administration could significantly prevent bone defects in GIO rats, suggesting its potential as a dietary supplement with beneficial effects against osteoporosis, which might involve the RANKL/OPG signaling pathway.

## Popular scientific summary

- Lactobacillus plantarum LP45 has beneficial effects for glucocorticoid-induced osteoporosis (GIO).
- LP45 prevents bone defects caused by GIO.
- A dietary supplement of LP45 would be helpful for osteoporosis.

Osteoporosis is associated with accumulative bone weakness that leads to risks of fracture and ranks the most common cause for broken bones in the elderlies (1). Guidelines of the World Health Organization (WHO) define osteoporosis as a hip bone density lower than that of an average young adult by 2.5 SDs (standard deviations), as determined by dual-energy X-ray absorptiometry. Osteoporosis is normally asymptomatic until fractures with none-to-little stress occur (2). The osteoporosis etiology could be attributable to increased bone loss and reduced bone mass. Because of the lower levels of estrogen in post-menopause women, bone loss often exacerbates in such individuals (3). Osteoporosis may also concur with other diseases and respective treatments, including anorexia, alcoholism, hyperthyroidism, oophorectomy, and kidney disease (4). Accumulated epidemiologic studies have identified several medicines, such as chemotherapy, antiseizure medications, inhibitors of proton pumps, glucocorticosteroids, and selective serotonin reuptake inhibitors, as contributing factors to the development of osteoporosis (5). In particular, glucocorticoids are being widely used in clinical practices as an anti-inflammatory treatment in various immune-related diseases, including organ transplantation and rheumatic disease (6). Based on the Global Longitudinal Study of Osteoporosis in Women (GLOW), 2.7~4.6% of women aged 55 years old or older are subjected to glucocorticoid therapy, which could result in a number of serious adverse effects that include glucocorticoid-induced osteoporosis (GIO), a typical reason for secondary osteoporosis. Hence, general assessments as well as protective measures for preventive purposes are recommended to all patients receiving glucocorticoids.

Probiotics are dietary supplements containing live non-pathogenic microorganisms, and in adequate amount can benefit the treatment as well as prevent pathological conditions (7, 8). Several genera of bacteria, for instance, Lactobacillus, Bacillus, Enterococcus, Bifidobacterium, and Escherichia, have been adopted as beneficial probiotics and commonly used in fermented products (e.g. beer, milk products, and meat), dietary supplements, or non-conventional products (toothpaste or ice cream). Recent reports implicated that the intestinal microbiota and physiology may also be important players in the regulation of bone health (9–15). Hence, probiotics, known to modulate the microbiota function and/or composition to improve intestinal health, may also benefit the health of bones. Multiple recent clinical investigations demonstrated the positive effects of probiotics on human skeletal health (16–18). Although these above studies have collectively shown the efficacy of probiotics in improving bone health, they were either investigating mixtures of strains or strains other than L45; no previous study has focused on L45. Moreover, the specific mechanism underlying probiotic benefits on bone health is yet to be fully understood, likely involving the immune system/cells.

In this study, we sought to study the potential beneficial effects of *Lactobacillus plantarum* LP45 on a rat model of GIO and to explore molecular pathways that might be involved in the observed beneficial effects of LP45.

## Materials and methods

### Animals and treatments

This study was conducted in conformity with the Guide for the Care and Use of Laboratory Animals by the Affiliated Yantai Yuhuangding Hospital of Qingdao University. All protocols obtained the approval from the Academic Committee on the Ethics of Animal Experiments of the Affiliated Yantai Yuhuangding Hospital of Qingdao University, and the approval number is KFG/45. Sprague–Dawley rats (male, 2-month-old) were accustomed to the local vivarium environment (humidity 67% and temperature 24–26°C) with ad lib access to water and rodent chows containing 0.8% phosphorus and 1.2% calcium. The rats were divided randomly into experimental groups (*n* = 8 per group) as follows: 1) Control (CON), rats were given distilled water (as vehicle control) via daily oral gavage for 8 weeks; 2) GIO, rats were administered with prednisone acetate at the dose of 6.0 mg/kg/day via oral gavage for 8 weeks; 3) LP45-L, rats were administered with prednisone acetate at the dose of 6.0 mg/kg/day and 5 × 10^8^ cfu/day LP45 via oral gavage for 8 weeks; 4) LP45-M, rats were administered with prednisone acetate at the dose of 6.0 mg/kg/day and 1 × 10^9^ cfu/day LP45 via oral gavage for 8 weeks; 5) LP45-H, rats were administered with prednisone acetate at the dose of 6.0 mg/kg/day and 2 × 10^9^ cfu/day LP45 via oral gavage for 8 weeks. LP45 strain was supplied by Hebei Inatural Biotech Co., Ltd (Hebei, China). Administration of prednisone acetate and LP45 was staggered with 2 h interval in the same day to improve absorption.

### Bone histomorphometry

Parameters of bone histomorphometric measurements followed the guidelines of the ASBMR Histomorphometric Nomenclature Committee (19) and previous methods (20, 21). The measured thickness (minus the cortical thickness [Ct.Th]) was multiplied by p/4. Structural parameters include bone volume (BV), tissue volume (TV), bone surface (BS), and Ct.Th. The microarchitecture parameters such as trabecular thickness [Tb.Th], number [Tb.N], and separation [Tb.Sp] were derived from measurements of areas and perimeters. Bone formation and resorption were evaluated by measuring osteoblast surface per bone surface (Ob.S/BS) and osteoclast surface per bone surface (Oc.S/BS).

### Bone mineral content and bone mineral density

The tibia and femur bones of rats were wrapped in saline-saturated gauze to prevent drying and stored at -20°C. When used for experiments, the bones were first thawed at room temperature, cleared of the residual muscles, and then moisturized by soaking in the saline solution. A Prodigy Dual-Energy X-ray Absorptiometry scanner (GE Healthcare, Little Chalfont, UK) was employed to scan the entire femoral bone mineral density (BMD) to obtain the bone mineral content (BMC) and bone area (BA, cm^2^), and BMD was quantitated as BMC/BA.

### Femoral biomechanics

Following BMD measurements, the femur bones were examined for mechanical properties in compression test or three-point bending test with the Material Testing System (MTS Systems, Eden Prairie, MN, USA). A 1 mm indenter was used to test the bones at a speed of 0.01 mm/s with a 15 mm span for femur, while force and deflection were being automatically recorded. The output parameters were maximum load (the maximum force the bone can resist, N), elastic load (the force needed to deform the bone, N), and fracture load (the force needed to produce bone fractures, N). The area moment of inertia and the stiffness coefficient (load–displacement curve slope, N/mm) were derived from these output parameters as well.

### Serum factor ELISA

Blood samples were stored in specimens’ tubes and left in a vertical position for 40–50 min at 25°C to completely clot. The serum was then obtained by 10 min centrifugation at 1,000 × g and frozen at -80°C for future biochemical assessments. Serum levels of receptor activator of nuclear factor kappa-B ligand (RANKL), osteocalcin, tartrate-resistant acid phosphatase 5 (TRAP5), and osteoprotegerin (OPG) were evaluated with commercial ELISA kits on the ELX800 Microplate Reader (Bio-Tek Instruments, Winooski, VT, USA) following the provided protocol.

### Real-time polymerase chain reaction

The total RNA from the bone marrow tissues was extracted with the TriZol reagent (Invitrogen, CA, USA) and checked for the RNA integrity using BioAnalyzer 2100 (Agilent, CA, USA), followed by reverse transcription to generate cDNAs using the High-Capacity DNA Reverse Transcription Kit (ThermoFisher, MA, USA). Real-time polymerase chain reaction (RT-PCR) was conducted on a HT7900 RT-PCR System (ABI, CA, USA) using the SYBR Green RT-PCR Master Mix (Tiangen, Beijing, China). Expression levels of genes of interest were normalized to the endogenous HPRT level and quantitated using the 2^-∆∆Ct^ method. Primers employed in the current study were as follows: RANKL forward 5’-ACGCAGATTTGCAGGACTCG-3’, reverse 5’-TTCGTGCTCCCTCCTTTCA-3’; osteocalcin F 5’-AAAGCCCAGCGACTCT-3’, R 5’-CTAAACGGTGGTGCCATAGAT-3’; TRAP5 F 5’-TTCTGTTCCAGGAGCTT-3’, R 5’-GCAGGCTGCTGGCTGAC-3’; OPG F 5’-TGGCACACGAGTGATGAATG-3’, R 5’-GCTGGAAAGTTTGCTCTTG-3’; HPRT F 5’-AAGCCTAAGATGAGCGCAAG-3’, R 5’-TTACTAGGCAGATGGCCACA-3’.

### Western blot

Bone marrow tissues were prepared using the T-PER Tissue Protein Extraction Reagent (ThermoFisher, MA, USA) supplemented with protease inhibitors to extract the proteins, following manufacturer’s instructions. The protein lysates were quantitated by BCA protein assay, and the same amount of total protein was separated through SDS-PAGE followed by being transferred to a polyvinylidene fluoride (PVDF) membrane. The membranes were then subjected to blocking using 1% BSA (bovine serum albumin, Sigma) and subsequently incubated with primary antibodies at 4°C overnight. Primary antibodies included RANKL, osteocalcin, TRAP5, OPG, and HPRT. HRP conjugated secondary antibodies were employed to reveal bands on an ECL-based imaging system.

### Statistical analysis

Results were expressed as mean ± SD, and analysis was conducted using the SPSS12.0 software for Windows (SPSS, Chicago, IL, USA). Variance analysis (ANOVA) with the Fisher’s PLSD test was employed to determine the statistical differences among groups. Probabilities (*P*) below 0.05 were regarded as significant.

## Results

### Effects of LP45 on tibia and femur bone structures of GIO rats

By the end of the 8-week treatment, we compared the bone histomorphometry of the tibia bone among all experimental groups. GIO significantly reduced BV/TV, Tb.Th, and Tb.N but increased Tb.Sp, when compared to the rats in the CON group (Table 1, *P < 0.05, compared to CON). However, LP45 administration in the GIO rats resulted in largely reversed trend in all of the above bone histomorphometric parameters in a dose-dependent fashion (Table 1, ^#^*P* < 0.05, compared to GIO). Likewise, bone histomorphometry of the femur bones of GIO rats was also defective in comparison with the CON group (Table 2, *P < 0.05, compared to CON), which could be dose-dependently rescued by the LP45 treatment (Table 2, ^#^*P* < 0.05, compared to GIO). Notably, LP45 administration also restored the body weight loss of observed in GIO rats (Supplementary Fig. 1). These results suggested that the 8-week oral LP45 administration could prevent the GIO-induced defects in bone structures in rats.

**Table 1:** Effects of LP45 on tibia bone structure of GIO rats

Index	CON	GIO	LP45-L	LP45-M	LP45-H
BV/TV (%)	13.9 ± 2.7	13.1 ± 2.9*	13.3 ± 2.5*	13.6 ± 3.1^#^	14.1 ± 2.9^#^
Tb.Th (μm)	57.8 ± 5.9	51.4 ± 6.2*	52.7 ± 7.1*	53.8 ± 6.7*^#^	56.1 ± 6.5^#^
Tb.Sp (μm)	331.5 ± 83.6	374.6 ± 71.4*	371.1 ± 68.2*	358.3 ± 73.4^#^	343.6 ± 75.9^#^
Tb.N (^#^/mm)	2.5 ± 0.4	2.1 ± 0.4*	2.2 ± 0.6*	2.2 ± 0.5*	2.4 ± 0.4^#^

BV, bone volume; TV, tissue volume; Tb.Th, trabecular thickness; Tb.Sp, trabecular separation; Tb.N, trabecular number; GIO, glucocorticoid-induced osteoporosis; LP, *Lactobacillus plantarum*. Data were shown in mean ± SD (*n* = 8 per group).

* *P* < 0.05, compared to CON;

# *P* < 0.05, compared to GIO.

**Table 2:** Effects of LP45 on femur bone structure of GIO rats

Index	CON	GIO	LP45-L	LP45-M	LP45-H
BV/TV (%)	21.1 ± 3.3	18.6 ± 3.2*	19.1 ± 3.2*	19.6 ± 3.4*^#^	20.4 ± 3.5^#^
Tb.Th (μm)	67.1 ± 6.3	57.3 ± 6.8*	59.1 ± 6.7*	64.3 ± 7.1*^#^	66.4 ± 7.0^#^
Tb.Sp (μm)	241.6 ± 61.5	287.4 ± 58.1*	274.6 ± 63.7*	268.8 ± 57.3*	248.3 ± 67.5^#^
Tb.N (^#^/mm)	3.3 ± 0.6	2.9 ± 0.5*	2.9 ± 0.5*	3.1 ± 0.6*^#^	3.2 ± 0.4^#^

BV, bone volume; TV, tissue volume; Tb.Th, trabecular thickness; Tb.Sp, trabecular separation; Tb.N, trabecular number; GIO, glucocorticoid-induced osteoporosis; LP, *Lactobacillus plantarum*. Data were shown in mean ± SD (*n* = 8 per group).

* *P* < 0.05, compared to CON;

# *P* < 0.05, compared to GIO.

### Effect of LP45 on femoral biomechanics of GIO rats

**Fig. 1:**
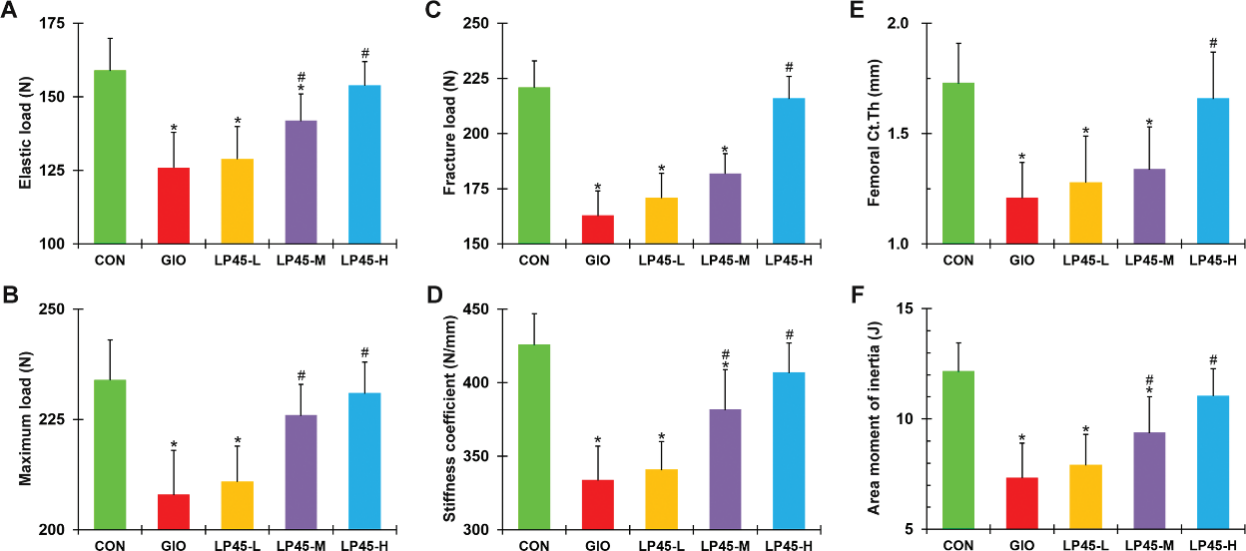
Effect of LP45 on femoral biomechanics of GIO rats. (A) Elastic load, (B) maximum load, (C) fracture load, (D) stiffness coefficient, (E) femoral cortical thickness (Ct.Th), and (F) area moment of inertia were measured on week 8 in CON, GIO, LP45-L, LP45-M, and LP45-H groups of rats (*n* = 8 per group). GIO, glucocorticoid-induced osteoporosis; LP, *Lactobacillus plantarum*. Data were expressed as mean ± SD. *P < 0.05, compared to CON; ^#^*P* < 0.05, compared to GIO.

We next evaluated the biomechanics, including elastic load, maximum load, fracture load, stiffness coefficient, Ct.Th, and area moment of inertia of the femurs. All of the above femoral biomechanical parameters were markedly reduced in the GIO group in comparison to the CON rats (Fig. 1, *P < 0.05, compared to CON) and were dose-dependently increased in the LP45-treated GIO rats (Fig. 1, ^#^*P* < 0.05, compared to GIO).

### Effects of LP45 on BMC, BMD, osteoblasts, and osteoclasts of the tibia and femur bones of GIO rats

BMC and BMD were also analyzed in the tibia and femur bones, both of which were substantially lower in the GIO group rats than the CON group (Tables 3 and 4, *P < 0.05, compared to CON). LP45 treatment could gradually increase BMC and BMD in a dose-dependent fashion in both the tibia and femur bones of GIO rats (Tables 3 and 4, #*P* < 0.05, compared to GIO). These data indicated that bone mineral deposition was disrupted by GIO, which could be rescued by LP45.

**Table 3:** Effects of LP45 on BMC, BMD, and osteoblast and osteoclast surfaces of the tibia bone of GIO rats

Index	CON	GIO	LP45-L	LP45-M	LP45-H
BMC (mg)	382 ± 46	317 ± 42*	326 ± 35*	352 ± 41*^#^	364 ± 38^#^
BMD (mg/cm^2^)	221 ± 26	194 ± 31*	202 ± 35*	211 ± 29*	227 ± 24^#^
Ob.S/BS (%)	8.9 ± 2.1	5.2 ± 2.4*	5.7 ± 2.8*	6.1 ± 3.1*^#^	8.2 ± 3.0^#^
Oc.S/BS (%)	0.62 ± 0.18	0.94 ± 0.31*	0.85 ± 0.29*	0.77 ± 0.33*^#^	0.68 ± 0.24^#^

BMC, bone mineral content; BMD, bone mineral density; Ob.S/BS, osteoblast surfaces per bone surface; Oc.S/BS, osteoclast surface per bone surface; GIO, glucocorticoid-induced osteoporosis; LP, *Lactobacillus plantarum*. Data were shown in mean ± SD (*n* = 8 per group).

* *P* < 0.05, compared to CON;

# *P* < 0.05, compared to GIO.

**Table 4:** Effects of LP45 on BMC, BMD, and osteoblast and osteoclast surfaces of the femur bone of GIO rats

Index	CON	GIO	LP45-L	LP45-M	LP45-H
BMC (mg)	437 ± 41	392 ± 36*	401 ± 29*	416 ± 32*^#^	424 ± 34^#^
BMD (mg/cm^2^)	287 ± 32	241 ± 36*	248 ± 43*	257 ± 41*	273 ± 38^#^
Ob.S/BS (%)	9.1 ± 2.4	4.7 ± 2.8*	5.1 ± 3.1*	6.9 ± 2.7*^#^	8.4 ± 3.3^#^
Oc.S/BS (%)	0.49 ± 0.23	1.08 ± 0.43*	0.93 ± 0.31*	0.84 ± 0.35*	0.57 ± 0.28^#^

BMC, bone mineral content; BMD, bone mineral density; Ob.S/BS, osteoblast surfaces per bone surface; Oc.S/BS, osteoclast surfaces per bone surface; GIO, glucocorticoid-induced osteoporosis; LP, *Lactobacillus plantarum*. Data were shown in mean ± SD (*n* = 8 per group).

* *P* < 0.05, compared to CON;

# *P* < 0.05, compared to GIO.

As bone mineral resorption is a pathological feature of bone loss-related diseases including osteoporosis, which involves the balanced action of both osteoblasts and osteoclasts (22, 23), we next examined these two types of cells in the tibia and femur bones, using Ob.S/BS and Oc.S/BS parameters. We discovered that Ob.S/BS was repressed, whereas Oc.S/BS was enhanced in both the tibia and femur bones of GIO rats (Tables 3 and 4, *P < 0.05, compared to CON), indicating that the osteoblast/osteoclast ratio was disrupted. Importantly, the LP45 treatment could significantly increase Ob.S/BS and reduce Oc.S/BS, thereby largely restored the GIO-disrupted osteoblast/osteoclast ratio in both the tibia and femur bones of GIO rats (Tables 3 and 4, #*P* < 0.05, compared to GIO).

### Effect of LP45 on serum factors of GIO rats

**Fig. 2:**
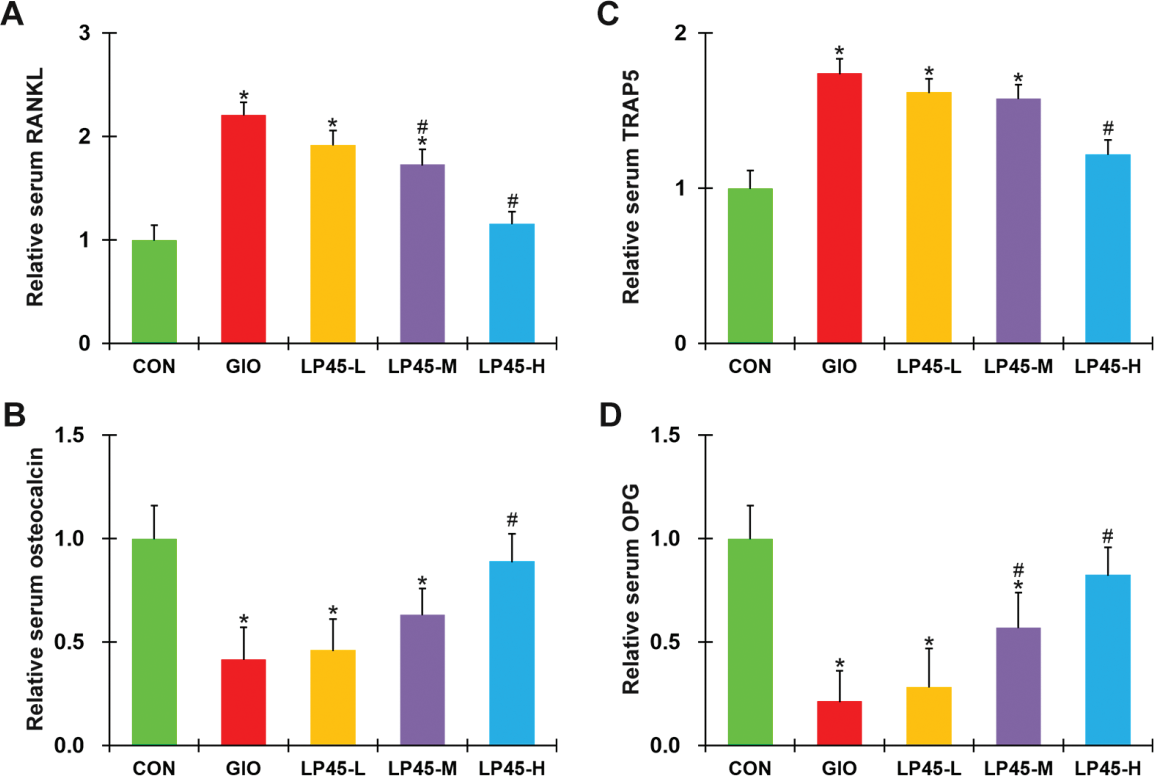
Effect of LP45 on serum factors of GIO rats. Serum levels of (A) RANKL, (B) osteocalcin, (C) TRAP5, and (D) OPG were measured on week 8 in CON, GIO, LP45-L, LP45-M, and LP45-H groups of rats (*n* = 8 per group) by ELISA. RANKL, receptor activator of nuclear factor kappa-B ligand; TRAP5, tartrate-resistant acid phosphatase 5; OPG, osteoprotegerin; GIO, glucocorticoid-induced osteoporosis; LP, *Lactobacillus plantarum*. Data were shown as relative to CON, in mean ± SD. *P < 0.05, compared to CON; #*P* < 0.05, compared to GIO.

As activation of the RANKL pathway has been reported to be closely involved in regulating osteoblast/osteoclast (22), we next assessed the serum levels of RANKL pathway factors, including RANKL and OPG, as well as osteogenic marker osteocalcin (24) and osteoclast marker TRAP5 (25), in the CON rats, GIO rats, and LP45-treated GIO rats by ELISA. We found that, comparing GIO rats with CON rats, serum RANKL was elevated (Fig. 2A), serum osteocalcin was reduced (Fig. 2B), serum TRAP5 was increased (Fig. 2C), and OPG was reduced (Fig. 2D), all of which indicative of defective osteoblast function and pathologically enhanced osteoclast function. On the other hand, LP45 treatment could dose-dependently reverse, to a large extent, the above serum factors, in the GIO rats (Fig. 2A–D, #*P* < 0.05, compared to GIO), consistent with a potential anti-osteoporosis property of LP45 that might involve the RANKL/OPG signaling pathway.

### Effect of LP45 on bone marrow factors of GIO rats

**Fig. 3:**
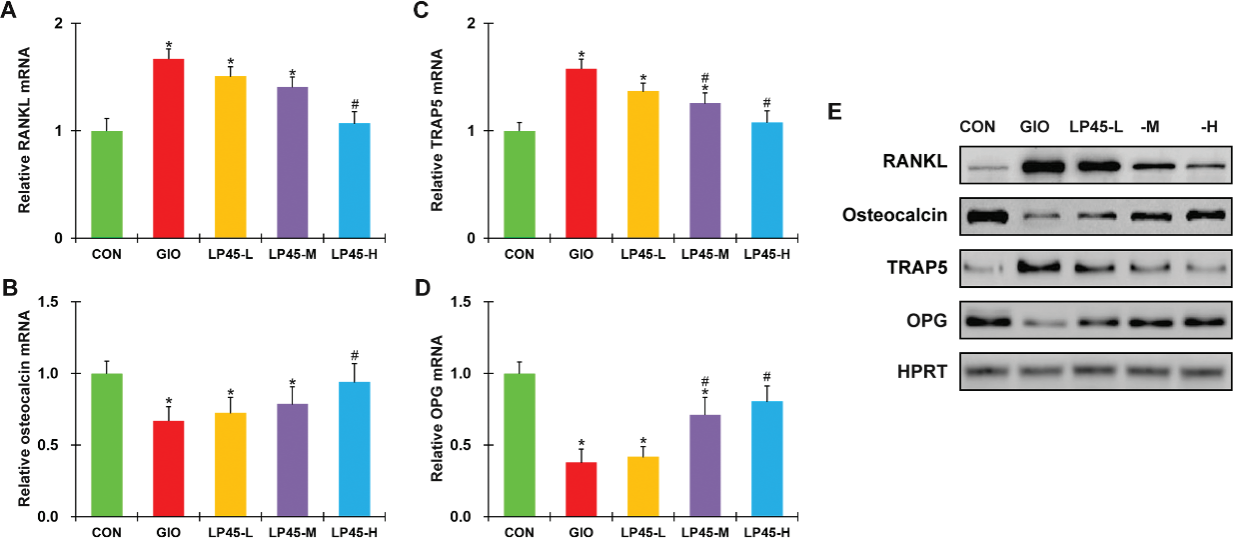
Effect of LP45 on the expression of tibia bone marrow factors of GIO rats. Transcript levels of (A) RANKL, (B) osteocalcin, (C) TRAP5, and (D) OPG, as well as (E) their protein levels were measured on week 8 in the tibia bone marrow of CON, GIO, LP45-L, LP45-M, and LP45-H groups of rats (*n* = 8 per group). RANKL, receptor activator of nuclear factor kappa-B ligand; TRAP5, tartrate-resistant acid phosphatase 5; OPG, osteoprotegerin; GIO, glucocorticoid-induced osteoporosis; LP, *Lactobacillus plantarum*. Data were shown as relative to CON, in mean ± SD. *P < 0.05, compared to CON; #*P* < 0.05, compared to GIO.

**Fig. 4:**
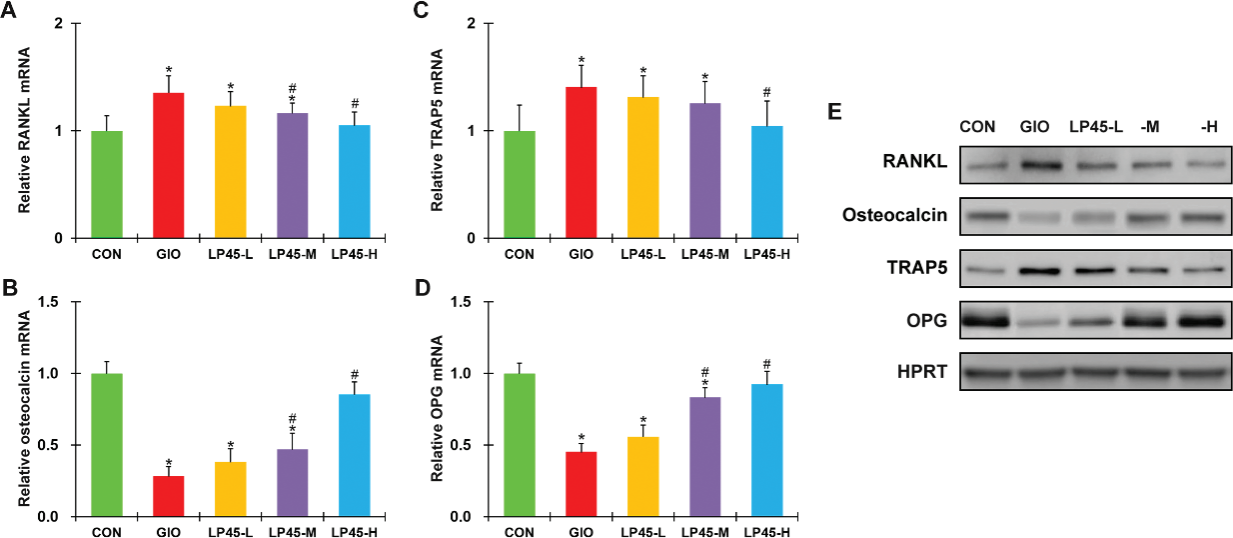
Effect of LP45 on the expression of femur bone marrow factors of GIO rats. Transcript levels of (A) RANKL, (B) osteocalcin, (C) TRAP5, and (D) OPG, as well as (E) their protein levels were measured on week 8 in the femur bone marrow of CON, GIO, LP45-L, LP45-M, and LP45-H groups of rats (*n* = 8 per group). RANKL, receptor activator of nuclear factor kappa-B ligand; TRAP5, tartrate-resistant acid phosphatase 5; OPG, osteoprotegerin; GIO, glucocorticoid-induced osteoporosis; LP, *Lactobacillus plantarum*. Data were shown as relative to CON, in mean ± SD. *P < 0.05, compared to CON; #*P* < 0.05, compared to GIO.

To further verify the observed anti-osteoporosis action of LP45 in the serum, we also extracted bone marrow samples from the tibia bones of all experimental animals and analyzed the same set of factors using RT-PCR and Western blot. The same defects were observed in the mRNA as well as protein levels of osteocalcin, TRAP5, RANKL and OPG in the GIO rat bone marrows compared with the CON rats (Fig. 3A–E, *P < 0.05, compared to CON), and these defects could be dose-dependently rescued by LP45 in the GIO rats (Fig. 3A–E, #*P* < 0.05, compared to GIO). Similarly, the exact same trend in the above factors could be reproduced in the femur bone marrow (Fig. 4A–E), further confirming that LP45 exhibited anti-osteoporosis effect in the pathological setting of GIO, which was mediated by the RANKL/OPG pathway.

## Discussion

In this study, we first established a rat model of GIO, which caused obvious defects in tibia and femur bone structures that could be rescued by oral administration of the probiotic strain LP45 in a dose-dependent fashion. Additionally, the GIO-induced reductions in BMC, BMD, Ob.S/BS, and increased Oc.S/BS were largely restored by LP45 administration dose-dependently. Osteoblasts represent the major cell type that promotes the development and remodeling of bones (26). There are direct interactions of osteoblasts with bone cells, hematopoietic stem cells, and osteoclasts (26), which contribute to maintain the balance between the resorption and the formation of bones (27). Osteoclasts are related to osteoblasts functionally, also participating in bone resorption (28, 29). In this study, we observed that in the GIO rats, the Ob.S/BS was decreased, whereas Oc.S/BS was increased, which are consistent with a similar report using a GIO rat model as well that reported reduced trabecular BVs (30). Furthermore, decreased osteoclasts and osteoblasts might be correlated with the suppressed turnover and formation of the bones caused by glucocorticoid as well (31). However, results from our current study have implied a few study limitations: 1) osteoclasts were increased in GIO rats in our study, rather than showing a decreasing trend according to previous reports (30, 31); 2) there was a discrepancy between the decreased osteoblasts and the increased expression of RANKL in the present study. Further investigations are required to address these limitations, e.g. whether they were due to variations in genetical background of the rats. Therefore, our data demonstrated the action of LP45 on rebalancing the ratio of osteoblast/osteoclast in the pathological condition of osteoporosis caused by glucocorticoid, which contributed to the alleviated osteoporosis symptoms in the GIO rat model.

Further evidences confirming the beneficial role of LP in rebalancing the proportion of osteoblast/osteoclast come from evaluating the osteogenic marker osteocalcin (24) and osteoclast marker TRAP5 (25). Osteocalcin is an osteoblast-specific gene that encodes an osteoblast-derived hormone involved in the regulation of insulin secretion (32). Osteocalcin, when inside of the osteoblasts, is carboxylated on three glutamine acid residues and then released into the bone extracellular matrix (33), where it modulates glucose metabolism in bones (34). TRAP5 belongs to a special group of iron-binding proteins, including uteroferrin and other purple AcPs, and normally can only be found in osteoclasts (35). High TRAP5 activity levels are observed in osteoclasts as well as in serum during bone resorption, with the bone resorption rate being correlated with the plasma concentration of TRAP5 (35). In the GIO rats, the reduced osteocalcin and elevated TRAP5 in both the serum and bone marrows clearly demonstrated enhanced bone resorption phenotype. The results showed that LP45 treatment could increase osteocalcin and suppress TRAP5 levels in the serum and bone marrows in the GIO rats, certainly supporting its beneficial action against pathological bone resorption by rebalancing osteoblast/osteoclast functions.

Importantly, we also found that LP45 dose-dependently restored the changes in RANKL pathway factors RANKL and OPG, in the serum as well as bone marrow of GIO rats, suggesting that the anti-osteoporosis role of LP45 in the pathological setting of GIO may involve the RANKL/OPG pathway. The RANKL/OPG system was initially revealed by parallel studies in the late 1990s and regarded as crucial to bone homeostasis through regulation of osteoclasts (36, 37). RANKL protein is a type II membrane protein consisted of a transmembrane domain and a C-terminal extracellular receptor-interacting domain (38). RANKL expression is readily inducible, under the regulation of multiple osteoactive factors such as glucocorticoids (39). It has been shown that RANKL may bind to both RANK (the functional receptor) and OPG (the decoy receptor) (36). OPG was initially identified as an osteoclastogenesis inhibitory factor (40) and primarily expressed by bone marrow stromal cells (41). OPG could be negatively regulated by glucocorticoids that disrupts bone homeostasis (42), consistent with current observations made in our GIO rat model. Moreover, the glucocorticoid-induced RANKL and repressed OPG expression could be readily reversed by LP45 administration in both the serum and bone marrows of the GIO rats, demonstrating the regulatory effect of LP45 on the RANKL/OPG pathway. Nevertheless, the exact molecular chain linking LP45 administration and RANKL/OPG remains elusive and warrants further investigation.

Indeed, our current work is an observation rather than mechanical study, and more investigations should be conducted to dive into the involved molecular mechanism, especially the involvement of the RANKL/OPG pathway. This is a limitation of our current work without mechanism study, which requires the use a cellular system to establish genetically modified model to study the potential casual relations. We are in the process of acquiring additional funding and resource to perform these experiments. Nevertheless, the observations made in our study could imply the potential utility of LP45 in a clinical setting for the treatment of GIO.

## Conclusion

To conclude, our present study hereby demonstrates that oral LP45 administration could significantly prevent pathologically enhanced bone resorption in GIO rats, suggesting its potential as a dietary supplement with beneficial effects against osteoporosis, which might involve the RANKL/OPG signaling pathway.
