# Supplementary material for: Lactobacillus plantarum LP45 inhibits the RANKL/OPG signaling pathway and prevents glucocorticoid-induced osteoporosis
Source: Food Nutr Res. 2023 Mar 24;67:10.29219/fnr.v67.9064. doi: 10.29219/fnr.v67.9064 (PMC10087339; doi:10.29219/fnr.v67.9064)
Supplement: Supplementary file 1 [file FNR-67-9064-s001.docx]

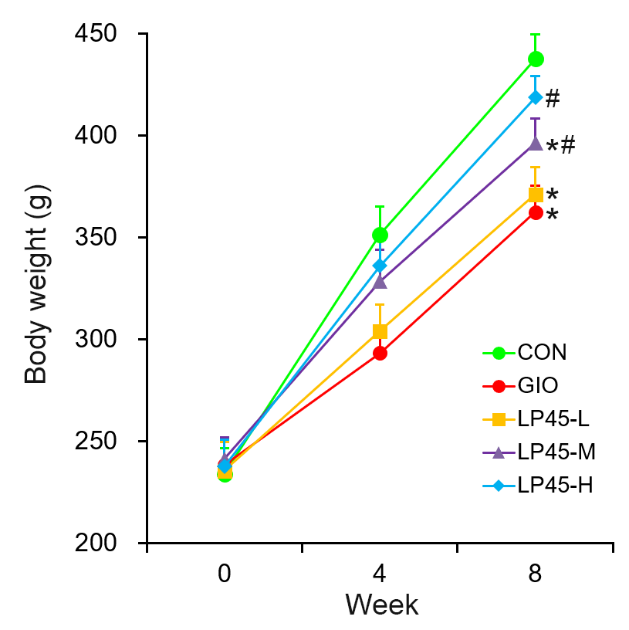


**Figure S1. Dose-dependent effect of LP45 on body weight of GIO rats.**

Body weight was measured on week 0, 4 and 8 in CON, GIO, LP45-L, LP45-M and LP45-H groups of rats (n=8 per group). Data were shown in mean ± SD. * p < 0.05, compared to CON; # p < 0.05, compared to GIO.
